# Determinants of health-related quality of life among human immunodeficiency virus positive (HIV-positive) patients at Ahmadu Bello University teaching hospital, Zaria, Nigeria- 2015

**DOI:** 10.1186/s12889-020-08659-9

**Published:** 2020-04-19

**Authors:** Bello Abdullahi Suleiman, Mohammed Yahaya, F. A. Olaniyan, A. G. Sule, M. B. Sufiyan

**Affiliations:** 1 Department of Family Medicine, Federal Medical Centre Katsina, Katsina State, Nigeria; 2 Nigeria Field Epidemiology and Laboratory Training Program, Abuja, Nigeria; 3 Department of Medical Microbiology & Parasitology, Usmanu Danfodiyo University, Sokoto, Nigeria; 4 Department of Family Medicine, Ahmadu Bello University Teaching Hospital, Zaria, Nigeria; 5 Department of Community Medicine, Ahmadu Bello University, Zaria, Nigeria

**Keywords:** Health, Quality of life, Determinants, HIV/AIDS, HIV-positive

## Abstract

**Background:**

The advent of Highly Active Antiretroviral Therapy (HAART) is associated with improved clinical and laboratory outcomes resulting in prolonged life and well-being of people living with Human Immunodeficiency Virus (PLHIV). However, the needs for life-long therapy, medications’ side effects and stigma have raised concerns about their quality of life (QOL). This study assessed the determinants of Health-related quality of life (HRQOL) among HIV-positive patients at Ahmadu Bello University Teaching Hospital (ABUTH) Zaria.

**Methods:**

We conducted a cross-sectional study of 353 HIV-positive adults on HAART attending the HIV clinic of ABUTH, Zaria. The participants were recruited into the study using a systematic sampling technique. Data on socio-demographics, medical parameters, QOL and family functionality were collected using structured, interviewer-administered questionnaire. The World Health Organization (WHO) Quality of Life HIV short form instrument (WHOQOL-HIV BREF) item and Family APGAR tool were respectively used in assessing the QOL and family functionality of the participants. We performed univariate, bivariate and multivariate analysis.

**Results:**

Mean age was 39.1(±10.9) years, 239 (67.7%) were females, 208 (58.9%) were Hausa-Fulani, 240 (68.2%) married and up to 210 (59.4%) had at least a secondary education. The overall mean scores on the scale of 4–20 for HRQOL were similar in three domains: environment domain 14.5(±2.8); social relationship 14.4(±3.1) and level of independence 14.4(±2.5). Lower scores were recorded in spirituality/religion/personal beliefs 12.3(±4.3). Identified determinants of HRQOL were spousal HIV- positive status (AOR = 3.37; CI; 1.46–7.74) and high family function (AOR = 2.57; CI: 1.51–4.39).

**Conclusion:**

Having highly functional family and having HIV-positive partner were the major determinants of HRQOL. Routine family counselling and strengthening the HIV social-support network should be incorporated into the routine patients’ care in HIV treatment centers.

## Background

Human Immunodeficiency Virus/Acquired Immune Deficiency Syndrome (HIV/AIDS) infection is a global pandemic, with cases reported from virtually every country. Worldwide, there is a decreasing trend of HIV infection and increase in survival of PLHIV. This is due to improved therapeutic and preventive measures. At the end of 2017, there were 36.9 million PLWHIV globally [1, 2]. AIDS-related deaths have been reduced by more than 51% since the peak in 2004 [1, 2]. In the year 2017, some 940,000 people died from AIDS-related illnesses worldwide, compared to 1.4 million in 2010 and 1.9 million in 2004 [1, 2].

The problem of HIV/AIDS has been more debilitating in sub-Saharan Africa. In 2017, there were 19.6 million PLHIV; (53%) in eastern and southern Africa, 6.1 million (16%) in western and central Africa [1, 2]. Nigeria is the most populous African country with the second largest burden of HIV/AIDS after South Africa [3, 4].

Studies have demonstrated the effectiveness of HAART in better clinical outcomes and the overall improvement of the QOL for PLHIV [5–7]. Even though with PLHIV being on life-long medications with the attendant consequences, this has brought out other issues that revolves around the psychosocial well being of these group of patients [8, 9]. All these indicators of QOL are now been considered as important factors in the overall management of PLHIV [8, 9]. HRQOL is a broad entity that denoted QOL based on patient’s clinical outcome [10, 11]. HRQOL further showcase the true definition of health as defined by the WHO [10, 11]. The psychosocial well-being of patients with chronic diseases is further enhanced by evaluation of QOL for this category of patient [9, 11]. Knowing the determinants of HRQOL would enable the patients, their families, healthcare providers as well as policy makers to devise relevant and holistic interventions to improve the general well-being and overall QOL of PLHIV.

We therefore assessed the determinants of HRQOL among HIV-positive adult patients attending HIV clinic of ABUTH, Zaria.

## Methods

### Study design

We conducted a hospital-based cross-sectional study at ABUTH Zaria, Kaduna state, Nigeria.

### Study setting

The study was conducted in the HIV/PEPFAR clinic of ABUTH, Zaria. The hospital is a 500 bed capacity, established tertiary institution. It serves as a referral centre for primary and secondary health institutions in northern Nigeria.

The HIV clinic was established in 2006 and provides services for the diagnosis, treatment and prevention of HIV, in addition to free counselling and testing. It has the capacity to accommodate five hundred patients per clinic day and has several units, including adult (Antiretroviral Therapy) ART clinic, Paediatrics ART unit, the Prevention of Mother-to-Child transmission (PMTCT) unit, HIV/TB co-infection unit, Pharmacy unit and a PEPFAR accredited laboratory. The clinic runs daily from Monday to Friday and has a vibrant/functional support group mainly supported by the social welfare department. The group helps members (PLHIV) in various ways such as continuous health education, mate-matching among the clients and financial empowerment of members for self-reliance through loans and trainings for small scale businesses.

### Study population

The study participants comprised of all adult patients (18 years or more) on HAART therapy at the HIV clinic of ABUTH Zaria, Kaduna State, Nigeria.

### Inclusion and exclusion criteria

All consented patients that attended the HIV clinic within the study period (March–May, 2015) were included. Patients that were severely ill, pregnant and those with psychiatric co-morbidities were excluded.

### Sample size estimation and sampling technique

A sample size of 353 was used for our study based on an expected proportion (*p* = 50%) of adults on ART with good HRQOL [12]. Systematic sampling technique was used to recruit the subjects from the sampling frame using a calculated sampling interval of 13. The first patient was randomly chosen out of the first 13 patients by balloting and the 7th patient was selected. Subsequently, the 20th, 33th, 46th and the n^th^ were serially recruited until the required sample size was obtained.

### Data type and source

An interviewer administered questionnaire was used in collecting the data. The structured questionnaire has four (4) main sections that include; the (socio-demographic variables), the medical parameters (clinical/laboratory), the adopted quality of life questionnaire [WHOQOL-HIV BREF] [9–18] and the APGAR SCORING system for assessing family functionality/support of the participants as sections one, two, three and four respectively. The WHOQOL-HIV BREF questionnaire has been widely used for QOL studies in PLWHIV, both locally and internationally [9–18].

The WHOQOL-HIV BREF is a 31 items tool distributed under 6 main domains comprising physical, social relationships, level of independence and spirituality domains with 4 items each. While the psychological and environmental domains have 5 and 8 items respectively. Each of the items was rated based on a 5-point Likert scale with 1 point as the lowest/negative perception and the highest/positive perception being the 5 points. The last 2 items in the tool measures overall perceived quality of life and general health perception. A mean of the overall scores of HRQOL domains was calculated and it was approximately 14.0, and this was used to categorize the subjects into two; high/good and low/poor QOL. A score of 14.0 and above signify high/good HRQOL while a score of less than 14.0 reflects poor/low HRQOL as used in the previous studies [12–14]. This method of categorization was adopted from previous (similar) studies done across the globe. The items in the tools were contextualized to the study area and translated to the local (Hausa) language [15–22]. Medical parameters were extracted from patients’ medical records. Family functionality/support variable was measured using a 5-item Family APGAR scale [15–22]. It was developed as a tool to measure a family member’s perception of family functioning. A simple screening test which gives a rapid review of the components of family functioning in a mnemonic fashion similar to the APGAR evaluation of the newborn. APGAR stands for **Adaptation**, **Partnership, Growth, Affection** and **Resolve**. Each is tense based on individual perception. It is a Questionnaire based tool that is scored 0, 1 and 2 respectively for “hardly ever”, “some of the time” and “almost always” to determine the individual’s perception of the respective issues represented by the acronym. Scores of 7–10 was considered as functional/supportive family while scores of 0–6 was considered dysfunctional/non-supporting family as used in previous studies [15–22].

## Data collection and analysis

Questionnaires were administered by researcher and the assistants. It was pretested at a similar site providing HIV/AIDS care within the state (Giwa General Hospital ART Clinic).

We conducted a univariate analysis to describe the mean, standard deviation, median, range, frequency and proportions. The domain scores in WHOQOL–HIVBREF were scaled in positive direction with higher score indicating good quality of life. A mean score (14.0) of HRQOL was used to place the study participants into two; poor/low HRQOL for score < 14.0 and high/good HRQOL for a score of 14.0 and above.

Bivariate analysis was carried out to evaluate the factors associated with HRQOL. However, variables which were found to be statistically significant at bivariate level and those with *p*-values < 0.10 in the overall HRQOL were considered for multiple logistic regression analysis to identify the determinants of HRQOL.

Finally, multivariate logistic regression analysis was fit in. Using the stepwise approach, only the variables significant at *p* < 0.05 were finally identified as determinants of HRQOL from all variables included in the logistic regression analysis. Adjusted odds ratios (AOR) of these significant variables with its corresponding 95% confidence intervals were also reported.

## Results

Most of the respondents 151 (42.8%) were between the ages of 35–44 years with a mean age of 39.1 (±10.9) years. Most were females 239 (67.7%) and Hausa-Fulani 208(58.9%) by ethnicity. The respondents were mostly Muslims 222 (62.9%) and 210 (59.4%) of them had western education up to secondary and tertiary levels. Equally, 240 (68.2%) of them were married and employed 266 (75.3%). In this study, 304 (86.2%) of the participants had HIV-positive partners (Table 1).

**Table 1:** Socio-demographic characteristics of HIV positive adult patients attending HIV Clinic at ABUTH Zaria, 2015 (*n* = 353)

Variables	Frequency (n)	Proportion (%)
**Age (years)**		
< 25	19	5.4
25–34	87	24.6
35–44	151	42.8
45–54	56	15.9
55–64	28	7.9
≥ 65	12	3.4
**Sex**		
Male	114	32.3
Female	239	67.7
**Ethnicity**		
Hausa-Fulani	208	58.9
Yoruba	13	3.7
Igbo	20	5.7
^a^Others	112	31.7
**Religion**		
Islam	222	62.9
Christianity	131	37.1
**Level of Education**		
Non-formal	18	5.1
Qur’anic/Islamiyya	80	22.7
Primary	45	12.7
Secondary	87	24.6
Tertiary	123	34.8
**Marital status**		
Single	31	8.8
Married	241	68.2
Divorced	37	10.5
Widow	44	12.5
**Employment status**		
Employed	266	75.3
Unemployed	61	17.3
Retired	10	2.8
Student	16	4.5
**Spousal HIV status**		
Positive	304	86.2
Negative	49	13.8
**Children’s HIV status (*n*** **= 322)**		
Positive	34	10.6
Negative	288	89.4

^a^Others: Bajju, Kataf, Jaba, Tiv, Idoma, Igala, Nupe

The mean scores of HRQOL of HIV/AIDS patients at ABUTH Zaria were highest in the environment 14.5 (±2.8), social relationship 14.4 (±3.1) and level of independence 14.4 (±2.5) domains. The lowest score was noted in the spirituality/religion/personal beliefs (SRPB) 12.3 (**±**4.3) domain. Higher mean scores of 15.2 (±3.9) and 15.0 (±3.9) were respectively recorded in the overall perception of HRQOL and general health perception (Table 2).

**Table 2:** Mean Scores of HRQOL of HIV positive adult patients attending HIV clinic at ABUTH Zaria by Domains, 2015 (*n* = 353)

HRQOL domains	Mean ± SD	Minimum-Maximum
Physical	13.3(±2.6)	4–20
Psychological	13.9(**±**2.4)	4–20
Level of Independence	14.4(**±**2.5)	4–20
Social relationship	14.4(**±**3.1)	4–20
Environment	14.5(**±**2.8)	4–20
Spirituality/religion/belief	12.3(**±**4.3)	4–20
Overall perception of HRQOL	15.2(**±**3.9)	4–20
Overall General health perception	15.0(±3.9)	4–20

**Fig. 1:**
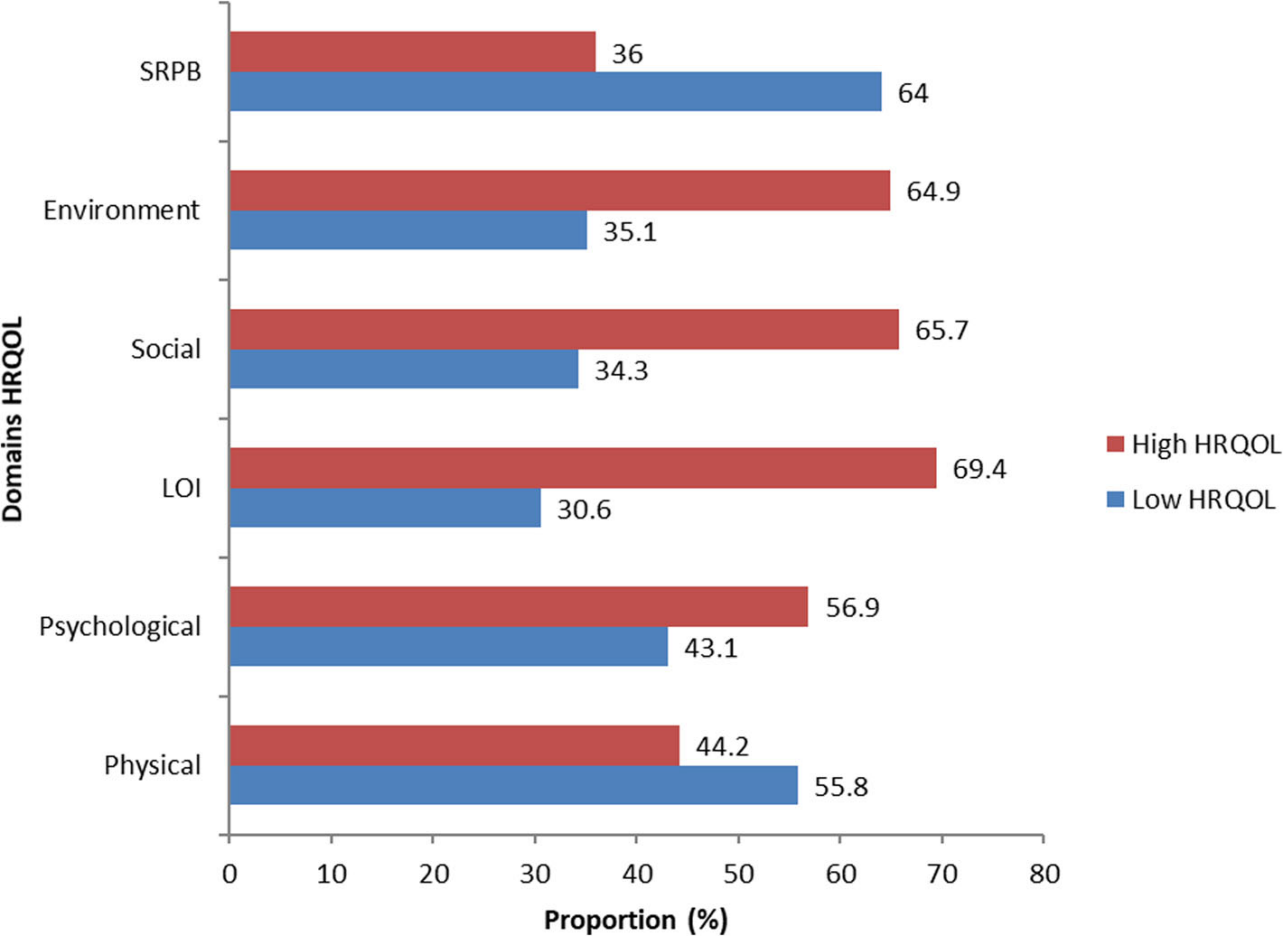
Proportion of HIV positive adult patients attending HIV clinic at ABUTH Zaria with High or Low HRQOL per domains, 2015 (*n* = 353)

The proportion of respondents with high HRQOL was highest in the level of independence domain (69.4%) followed by social relationship domain (65.7%), environment domain (64.9%), and psychological domain (56.9%). The proportion of those with low HRQOL was highest in the spirituality/religion/personal belief domain (64.0%) and physical domain (55.8%). The proportion of respondent having High or Low HRQOL scores are shown in Fig. 1.

Bivariate analysis indicated a statistically significant relationship between HRQOL and marital status (OR: 1.81, CI: 1.12–2.94), educational status (OR: 1.68, CI: 1.07–2.63), spousal HIV status (OR: 0.35, CI: 0.16–0.77), disease stage (OR: 2.10, CI: 1.13–3.73) and family functionality of the patients (OR: 0.38, CI: 0.23–0.61)) (Table 3).

**Table 3:** Socio-demographic characteristics, Medical parameters and HRQOL among HIV positive adult patients attending HIV clinic in ABUTH Zaria, 2015 (*n* = 353)

***Variables***	***HRQOL***		OR (CI)	***P-value***
	***High HRQOL*** n (%)	***Low HRQOL*** n (%)		
**Age (years)**				
< 35	35 (33.0)	71 (67.0)	0.84 (0.53–1.34)	0.475
≥ 35	95 (38.4)	152 (61.6)		
**Sex**				
Male	45 (39.5)	69 (60.5)	0.86 (0.54–1.36)	0.526
Female	86 (36.0)	53 (64.0)		
**Religion**				
Islam	79 (35.6)	143 (64.4)	0.84 (0.54–1.31)	0.440
Christianity	52 (39.7)	79 (60.3)		
**Marital status**				
Currently Married	100 (41.4)	141 (58.6)	**1.81 (1.12–2.94)**	**0.015**
Not Married	31 (28.1)	81 (71.9)		
**Educational status**				
Secondary/Tertiary	88 (41.9)	122 (58.1)	**1.68 (1.07–2.63)**	**0.024**
Qur’anic/Primary/None	43 (30.1)	100 (69.9)		
**Occupation**				
Employed	102 (38.4)	164 (61.7)	1.20 (0.74–2.07)	0.401
Not Employed	29 (33.3)	58 (66.7)		
**Spousal HIV status**				
Positive	121 (39.7)	183 (60.3)	**0.35 (0.16–0.77)**	**0.007**
Negative	9 (18.6)	40 (81.4)		
**Disease Stage**				
Not Advanced Disease	114 (40.1)	170 (59.9)	**2.10 (1.13–3.73)**	**0.017**
Advanced Disease	17 (24.6)	52 (75.4)		
**Comorbidities**				
Present	7 (24.1)	22 (75.9)	0. 271 (0.06–1.23)	0.070
Absent	124 (38.3)	200 (61.7)		
**CD4 Count (cells/mm**^**3**^**)**				
< 500	72 (34.0)	140 (66.0)	0.71 (0.46–1.11)	0.133
≥ 500	59 (41.8)	82 (58.2)		
**HAART Regimen**				
First line	111 (39.1)	173 (60.9)	1.60 (0.89–2.79)	0.119
Second line	20 (29.0)	49 (71.0)		
**Duration on Therapy (years)**				
< 5	29 (30.5)	66 (69.5)	0.65 (0.39–1.08)	0.097
≥ 5	88 (41.1)	126 (58.9)		
**Family Functionality**				
Functional Family	101 (44.9)	124 (55.1)	**0.38 (0.23–0.61)**	**< 0.001**
Dysfunctional Family	30 (23.4)	98 (76.6)		

Logistic regression analysis revealed partner’s HIV-positive status (AOR = 3.37, CI:1.46–7.74) and high family function (AOR = 2.57, CI:1.51–4.39) as the only independent predictors/determinants of HRQOL (Table 4).

**Table 4:** ^a^Determinants of HRQOL among HIV positive adult patients attending HIV clinic at ABUTH Zaria, 2015 (*n* = 353)

Variables	AOR (CI)	***p***-value
**Age (Years)**		
0–34	1.06 (063, 1.78)	0.820
≥ 35	1.00	
**Sex**		
Male	1.29 (0.76, 2.19)	0.337
Female	1.00	
**Religion**		
Islam	0.98 (0.58, 1.63)	0.930
Christianity	1.00	
**Marital status**		
Married	1.19 (0.64, 2.21)	0.580
Not married	1.00	
**Educational status**		
Secondary/Tertiary	1.20 (0.69,2.11)	0.520
Qur’anic/Primary/None	1.00	
**Occupation**		
Employed	1.23 (0.66, 2.31)	0.513
Not employed	1.00	
**Spousal HIV status**		
Positive	^**b**^**3.37 (1.46, 7.74)**	**0.004**
Negative	1.00	
**Disease Stage**		
Not Advance Disease	0.80 (0.41, 1.58)	0.525
Advance Disease	1.00	
**Comorbidities**		
Present	0.29 (0.06, 1.43)	0.124
Absent		
**Family Functionality**		
Functional Family	^**b**^**2.57 (1.51, 4.39)**	**< 0.001**
Dysfunctional Family	1.00	

^a^ Multivariate- logistic regression analysis. ^b^ Major determinants of HRQOL

## Discussion

In this study, majority of the participants were females signifying a high and disproportionate affectation of women by HIV. These findings were respectively consistent with findings in Ibadan (62.0%), Kogi (62.7%) and Sagamu (69.1%) [15–17]. Women were more vulnerable to HIV infection than men during unprotected sexual intercourse, because of larger surface areas exposed to contact during intercourse and because the females are the recipient of infected semen, as well as possible micro trauma in their genital tract during sexual activity. Likely explanation could also be due to high levels of polygamy, early marriage, early sexual debut, female genital mutilation and low girl child education in the study environment.

We found few participants with post-secondary education and this was far less compared to the findings in Osogbo and China respectively, where up to 65.4 and 44% had post-secondary education [18, 23]. This disparity may be due to low literacy level, low socio-economic status, high drop-out rate especially due to early marriage and low girl child education in the study area.

The study revealed that most of the subjects had HIV-positive partners and only one-tenth of them had HIV-positive children. Possible explanations could be due to increased number of marriages (mate-matching) among the HIV-postive patients facilitated by the HIV-support group in the facilty. Furthermore, scaled up preventive programs especially the prevention of mother-to-child transmission (PMTCT) services may be responsible for the low number of HIV-positive children.

Our study revealed higher mean scores of HRQOL in the environment domain. This was followed by the social relationship domain and then the level of independence domain. Lower mean scores were recorded in the psychological and physical domains. The spirituality/religion/personal belief domain had the lowest score. These findings were contrary to similar study from Kogi state, north-central Nigeria, in which the participants reported better QOL scores in the psychological, physical and spirituality domains, but lower scores in the environment and social domains [16]. Folasire in Ibadan reported higher mean scores in the physical, psychological and environment domains and lower scores in the social domain [15]. Our findings were in congruence with report from Henan- province, China where higher mean scores were recorded in the social and environment domains followed by the psychological domains [23]. In this study, the high domain scores recorded in the environment, social and level of independence reflect on the quality of care offered to these patients. Similarly factors like reduced stigma, non-discrimination, and increased acceptability in the community as well as good social support networks and other clinical interventions such as on-going psychotherapy might have played a role in improving the quality of life of these patients particularly in these domains [24]. Generally, in a country like Nigeria, it has been perceived that people tend to be more spiritual and religious only when confronted with issues that are beyond them [15]. The findings in this study did not agree with this belief. Lowest mean scores in the spirituality/religion/personal belief domains may be due to the wrong religious perception of HIV infection in the study area. A study on the influence of religious beliefs on HIV stigma, disclosure, and treatment attitudes in Tanzania indicated that shame-related HIV stigma is strongly associated with religious beliefs such as the belief that HIV is a punishment from God or that PLWHIV have not followed the word of God [25]. Similarly, in this study environment, people see HIV/AIDS patients as sexually promiscuous that go against the teachings of their religion. These may contribute to the patients’ non-disclosure of their status due to fear of blames and discrimination, and fear of their future or even death [11].

In this study, more than half of participants had high HRQOL scores in the environment, social, level of independence and psychological domains. This is an inverse picture of the Bangladesh study [14]. The highest proportion of individuals had low HRQOL in the physical and spirituality/religion domains suggesting the severest impact of HIV extended across physical and spiritual/religious aspect of HRQOL. This is expected as people with HIV/AIDS often experience physical inability, derogation, stigmatization, discrimination and marginalization [24].

This study also identified that couples with HIV-positive partners have better HRQOL compared to those with HIV-negative partners. A significant relationship was found between partners’ HIV status and HRQOL. This is contrary to the report from China on a study of QOL in sero-discordant couples [23]. It can be explained by the kind of support, accommodation and care the patients enjoy from their fellow HIV-positive partners without fear of blames, stigma or discrimination. Similarly, regular clinic attendance and participation in the support group activities is more in participants with positive partners.

Our study noted asymptomatic patients with less severe disease and no co-morbidity to have higher/better HRQOL scores compared to their symptomatic counterparts. This was similar to earlier studies in which significant differences in HRQOL scores were observed between patients who were asymptomatic, symptomatic, as well as those with AIDS [15, 23, 26]. Akinboro and colleagues reported a significant relationship between HRQOL and CD_4_ count, disease clinical stage, presence of TB co-morbidity and ART treatment in Osun [18]. Conversely, Sagamu study reported no relationship between HRQOL and all the patients’ medical parameters [27]. The explanation of these findings is that those who are sick (advance disease) are burdened with severe symptoms of the disease, which in turn, impair their HRQOL. It is believed that the occurrence of two severe diseases can impact negatively on the QOL of the patients [28].

Most of the participants with dysfunctional (non-supportive) family had low HRQOL. Several studies had reported the significance of family support in improving the HRQOL of HIV/AIDS patients [29–31]. Odili and colleagues in a similar study in Benin-Nigeria, had reported a significant difference observed in all domains among respondents with family support compared to those without family support [32]. A well-functioning supportive family could support the patient in many aspects such as physically, socially, psychologically, emotionally and even spiritually.

This study identified partners’ HIV- positive status and high family function as independent predictors of HRQOL among HIV-positive adult patients attending HIV clinic at ABUTH, Zaria. Many studies identified individuals that were married, in relationship or staying with an adult to have better HRQOL when compared to those that were separated, single or had lost their spouses [33–37]. It is well known that the family setting provides safety, security and financial support. Therefore, those who were living with their family will likely enjoy better social support, closer interpersonal relationship and satisfactory sexual activity which in turn impact positively on their HRQOL [36, 37]. Several studies had highlighted the relevance of family/social support in determining the HRQOL of PLWHIV [29–32].

### Limitations

As this study is cross-sectional one, it cannot establish causality of the associations between the outcome variable and independent variables. Inclusion of only HIV-positive patients on HAART is not likely to provide a true picture on the determinants of HRQOL among HIV positive patients, as the study was performed prior to Universal Test and Treat (UTT) recommendations adopted in Nigeria. Therefore, updated assessment in the context of UTT is needed to overcome this limitation.

## Conclusions

The study identified married subjects, with secondary or tertiary level of education, HIV-positive partners, less severe disease and functional family to have better HRQOL. It also reported higher mean scores for HRQOL in the environment, level of independence and social domains with the lowest scores in the spiritual/religion/personal belief domain. The major predictors of HRQOL in this study were having HIV-positive partner and highly functional family. We therefore recommend routine family counselling and strengthening the HIV social-support network should be incorporated into the routine patients’ care in HIV treatment centers.

## Data Availability

The collected data for this study are available at the hands of the corresponding author and the data are not shared to third party to maintain confidentiality of patients’ data. Clinical, laboratory and ART data were collected through reviewing records from ART cards and individual follow-up records using a structured questionnaire.

## Abbreviations

ART: Antiretroviral therapy
CI: Confidence interval
ABUTH, Zaria: Ahmadu Bello University Teaching Hospital, Zaria
HAART: Highly active anti-retroviral therapy
HRQOL: Health related quality of life
PLHIV: People Living with HIV
QOL: Quality of life
WHO: World Health Organization
WHOQOLHIV-BREF: World Health Organization quality of life of HIV specific instrument brief
